# Supplementary material for: Are carbon emissions trading and green financial instruments synergistic? -Comprehensive quantitative research based on content analysis
Source: PLoS One. 2024 Mar 7;19(3):e0298601. doi: 10.1371/journal.pone.0298601 (PMC10919601; doi:10.1371/journal.pone.0298601)
Supplement: S1 Data — (ZIP) [file pone.0298601.s001.zip › Data/地级市Z_F工作报告.zip!/▒▒╛⌐/└┤╫╘╣1⁄2╓┌║┼í╛┬φ┐╦╩2╛▌═°í┐_2012.docx]

——2012年1月12日在北京市第十三届人民代表大会第五次会议上

北京市市长 郭金龙

各位代表：

　　现在，我代表北京市人民政府，向大会报告政府工作，请予审议，请市政协各位委员提出意见。

　　一、2011年工作回顾

　　过去一年，在党中央、国务院和中共北京市委的坚强领导下，全市人民隆重纪念中国共产党成立90周年，深入贯彻落实科学发展观，坚决贯彻中央大政方针和对北京市工作的重要指示精神，加快实施“人文北京、科技北京、绿色北京”战略，坚定信心，团结奋斗，克服困难，推动首都科学发展取得新的成就，实现了“十二五”时期良好开局。

　　初步核算，全市地区生产总值达到1.6万亿元，比上年增长8.1%；地方公共财政预算收入达到3006.3亿元，增长27.7%；城镇登记失业率为1.39%，城镇居民人均可支配收入32903元，农村居民人均纯收入14736元，分别实际增长7.2%和7.6%。

　　一年来，我们坚持以科学发展为主题，以加快转变经济发展方式为主线，认真落实市十三届人大四次会议决议，重点做了以下几方面工作：

　　(一)坚决贯彻中央宏观调控政策，保持经济平稳较快发展

　　坚持落实扩大内需的战略方针，努力挖掘内需增长潜力。进一步完善政策、优化环境，建立促进消费责任制，推动消费增长向多点支撑转变。在机动车销售量下降44.3%、商品住宅销售面积下降13.9%的情况下，社会消费品零售额增长10.8%。发挥政府投资引导放大作用，拓宽投融资渠道，保证了重点工程、重大项目的资金需求，投资结构进一步优化。全社会固定资产投资增长13.3%，其中，政策性住房投资增长94.9%，民间投资增长14.2%，高技术行业投资增长73%。

　　坚持房地产调控，制定实施15条具体措施，率先作出新建普通住房价格“稳中有降”的承诺，促进房地产结构调整和平稳健康发展。新建普通住房成交均价同比下降11.3%，居民首购比重接近90%，投机投资性购房得到有效遏制。加大住房保障力度，注入100亿元财政资金成立了市保障性住房建设投资中心，优先保障用地需求，新建收购各类保障房23万套、竣工10万套，超额完成年度任务。完善公共租赁住房政策，将城市低收入家庭、新就业职工和外地来京工作一定年限人员纳入保障范围，全年配租公租房1万套。

　　坚持扶生产、保供应，多次出台具体调控措施，安排价格调节资金5亿元，切实控制物价过快上涨。全力抓好“菜篮子”工程，划定基本菜田57.9万亩，新增基本菜田5.5万亩，新建外埠蔬菜生产基地4万亩，加强与供应地合作，积极推动产销对接，减免大型批发市场、农贸市场进场费和摊位费，降低零售环节经营成本，农副产品供应保持稳定。开展专项整治和重点稽查，规范房屋租赁价格。严厉查处价格违法违规行为，有效维护了市场和价格秩序，居民消费价格月度涨幅已连续4个月回落，全年同比上涨5.6%。

　　(二)深入推进经济结构调整，加快转变经济发展方式

　　中关村国家自主创新示范区建设取得新成效。认真落实“1+6”系列政策，创新平台开始运行，481家单位实施了股权和分红激励，261个项目开展了科技成果处置权、收益权改革。建立部市会商机制，实现重大事项联合审批。制定实施优化产业布局、促进科技成果转化和产业化等指导意见，统筹政府资金100亿元支持了300余个重大科技成果产业化项目。发布中关村科学城发展规划，启动建设了航天科技创新园等37个项目。加快未来科技城建设，15家中央企业项目全部开工。建立军民融合的创新发展机制，推动军转民技术、军民两用技术产业化。加强人才特区建设，实施10项具体扶持政策，开辟吸引高端人才绿色通道，引进国内外高层次人才1962名。示范区规模以上企业总收入达到1.9万亿元、增长20.9%，收入过亿元企业达到1500家左右，新一代移动通信、软件、集成电路、生物医药等产业集群效应初步显现。推进实施“科技北京”行动计划，合作建立了超级云计算中心、国家技术转移中心，新增科技条件平台130个，认定高新技术企业1285家。发布实施首都标准化战略纲要，深入推进知识产权先导工程，全市技术合同成交额、发明专利授权量分别增长19.7%和41.7%，科技创新呈现强劲势头。

　　产业结构调整迈出新步伐。加快发展生产性服务业，金融中心城市功能进一步增强，信息服务、商务服务、科技服务带动作用更加突出。加快文化创意产业集聚区建设，支持文化企业重组、改造和上市，预计文化创意产业总收入超过9000亿元，增长20%以上。切实做好旅游促进工作，推动旅游与文化、体育等产业融合，国内外旅游人数达到2.1亿人次，旅游总收入3216.2亿元。第三产业比重为75.7%。制定加快培育和发展战略性新兴产业的实施意见，确定首批160个新兴产业重大项目，与15家中央单位签订战略合作协议，一批重点项目建成投产，规模以上高技术制造业、现代制造业增加值分别增长10.4%和10.6%。认真做好节能减排工作，率先实行能耗强度和能源消费总量双控机制，关闭高耗能、高耗水、高污染企业45家，完成4.7万户农民住宅抗震节能改造，更新淘汰老旧机动车22.4万辆，新增纯电动车1100辆，单位地区生产总值能耗、水耗和主要污染物排放量继续全面下降。

　　农村改革发展稳步推进。继续深化农村各项改革，集体林权制度主体改革任务基本完成，87.3%的集体经济组织完成产权制度改革。开展集体土地确权登记颁证、新型农村社区建设和“一事一议、财政奖补”试点，初步建立起农村基础设施运行管护机制。有序推进现代农业示范创建，籽种农业、观光休闲农业收入分别增长24.3%和20.9%，农业功能不断拓展。加强农田水利建设，完成30万亩农业基础建设及综合开发，新增和改善节水灌溉面积15万亩。制定实施推进农业现代化和郊区工业化、城镇化的意见，加快新农村建设步伐，设立小城镇发展基金，加快培育特色产业。积极促进农民增收，继续实施共同致富行动计划，农民收入增速连续三年快于城镇居民。

　　区域协调发展进一步加强。全面抓好城乡结合部城市化工程，制定促进集体产业发展、整建制农转居的意见，完善一道绿隔政策，50个重点村拆迁任务基本完成，回迁安置房建设加快推进。全力实施聚焦通州战略，进一步完善新城建设领导体制，重大市政基础设施建设进展顺利，重点产业项目加快落地。深入实施城南行动计划，三年建设任务完成了70%。制定加快西部地区转型发展的实施意见，永定河“四湖一线”生态景观带建成开放。生态涵养发展区环境友好型产业加快培育，沟域经济项目全面启动，7574名山区农民实现搬迁。“六高四新”功能区、南北两大产业带建设加快推进，聚集优质要素、承载产业发展的功能进一步增强。

　　(三)加大城乡建设管理力度，夯实可持续发展基础

　　交通治堵取得一定成效。加快轨道交通建设，3条线路顺利开通，新增运营里程36公里，总里程达到372公里。加大城市次干道、支路建设力度，完成15项微循环、110项疏堵工程建设。在确保安全前提下，进一步缩短地铁发车间隔，平均运力提高10%。启用京通快速路公交通勤走廊，实现市郊铁路公交化示范运营，开通一批“袖珍公交”和社区通勤快车。

　　实行机动车限购，机动车保有量同比少增61.7万辆。加强交通秩序管理，调整中心城区非住宅区停车收费价格，开展停车服务季活动。高峰时段路网平均运行速度提高12%左右，公交出行比例达到42%，小汽车出行比例首次下降。

　　生态环境质量继续提升。加快鲁家山等生活垃圾处理设施建设，在2454个城乡社区开展分类达标试点，生活垃圾产生量连续两年下降，焚烧、生化处理比例均提高到15%，再生资源回收量增长8%。加大污水处理设施建设改造力度，全市污水处理率达到82%，再生水利用量7.1亿吨。建成5个新城滨河森林公园、10个郊野公园、10个城市休闲公园，新增造林绿化面积25万亩，全市林木绿化率达到54%。实施清洁空气行动计划，空气质量二级和好于二级天数达到78.4%，一级天数达到74天、增长39.6%，实现了连续13年持续改善。制定进一步加强水务改革发展的意见，落实水务投资各项政策，成立南水北调工程投资中心，水务基础设施建设明显提速。

　　城市精细化管理水平不断提高。开展“精细管理、美化市容”工作，实施环境秩序“三个百日整治”，加强市容环境卫生综合考评，解决了一批群众反映强烈的突出问题。启动95个老旧小区电网改造，完成260公里架空线入地，初步实现重点区域无线局域网全覆盖。做好水电气热供应，东南热电中心、怀密天然气干线等工程建成投入使用，资源能源保障能力进一步增强。积极应对局地暴雨等极端天气影响，提高了道路排水设施设计标准，加大雨水系统改造和雨洪利用力度，城市运行平稳有序。

　　(四)全面加强社会建设，促进经济社会协调发展

　　在应对国内外复杂经济形势，特别是物价高位运行的困难情况下，我们更加注重保障和改善民生，切实解决人民群众最关心、最直接、最现实的利益问题。

　　就业和社会保障工作不断加强。市级财政投入293.2亿元，为群众办的30件重要实事全面落实。积极做好就业困难地区和困难群体帮扶工作，基本完成首钢停产职工分流安置任务，把城市化地区农村劳动力纳入城镇失业登记范围，加强职业技能培训，城镇新增就业44.7万人。推动制度整合衔接，把失业人员纳入职工基本医疗保险，开展转移就业农村劳动力参加职工社会保险试点，实现市级公费医疗制度与职工医保制度并轨、在职职工养老和医疗保险跨地区转移接续、工伤和生育保险制度全覆盖。健全社会保障相关待遇标准与物价上涨挂钩联动机制，年内两次提高标准，最低工资提高20.8%，农村低保最低标准提高61.9%，企业退休人员月平均养老金提高10%以上，向退休人员、征地超转人员和困难群众发放一次性生活补贴，累计投入财政和社保资金60多亿元、惠及310多万城乡居民。加快适度普惠型社会福利体系建设，统筹5.2亿元支持养老服务事业发展，养老机构床位总量达到8.2万余张。

　　教育、卫生改革发展成效显著。市级财政相关投入316.4亿元，增长28.6%。编制中长期教育改革和发展规划纲要，启动教育发展重大项目和改革试验，推进义务教育均衡发展改革试点。基本完成中小学校舍安全工程。制定实施学前教育三年行动计划，提高公用经费定额标准，投入6亿元支持改善办园条件，新增入园名额3.3万个。深化医药卫生体制改革，分类开展公立医院改革及医保付费制度改革试点，五项重点改革任务顺利完成。推广家庭医生式服务、双休日门诊、优质护理等服务模式，预约挂号平台覆盖了全部66家三级医院。全面实施基本公共卫生服务项目，积极应对医疗需求增长，群众看病就医条件进一步改善。

　　文化改革创新取得新进展。精心组织纪念中国共产党成立90周年宣传教育工作，提炼了“北京精神”，成为新时期践行社会主义核心价值体系的创新之举。开展“做文明有礼的北京人”等系列文明创建活动，思想道德和精神文明建设取得新成效。加强文化改革发展顶层设计，研究制定了相关政策体系。成立五大文化联盟，文艺院团和非时政类报刊出版单位转企改制扎实推进，都市报管理体制进一步理顺。开展网络实名制试点，开通了“北京微博发布厅”，制定实施了微博客发展管理规定。积极推进文化惠民工作，丰富基层群众文化生活，“一街一品”特色渐次形成。编制历史文化名城保护建设规划，历史文化建筑修缮工程稳步推进。发布全民健身实施计划，完善公共体育服务体系，群众体育活动广泛开展，首都国际体育影响力进一步提升。

　　社会管理创新深入推进。制定加强和创新社会管理、全面推进社会建设的意见，编制社会建设规划纲要。开展社区规范化建设及和谐社区创建活动，启动“六型社区”建设，推动社区基本公共服务全覆盖试点。新建“一刻钟社区服务圈”243个，累计达到409个，惠及620万居民。城市网格化管理深入推进，村庄社区化管理试点成效显著。基本完成城市社区服务站建设，建成1700个农村社区服务站。推动组织机构代码实名制管理，全面实现市级“枢纽型”社会组织分类管理，四类社会组织实行直接登记，全市1249座商务楼宇基本实现社会服务管理全覆盖。统筹做好人口与计划生育工作，总结推广人口服务管理成功经验，制定了支持产业优化升级、加强业态调整的工作意见，积极探索人口规模有序调控新途径，推进中心城区功能和人口疏解，人口基层基础工作进一步加强。

　　(五)着力推进改革开放，为首都转型发展注入新的动力和活力

　　重点领域和关键环节改革不断深化。行政管理体制改革继续推进，成立了市旅游委、市医院管理局、市预防腐败局，调整市城管执法局隶属关系，基本完成乡镇机构改革。完善全口径政府预算体系，加大财政预决算、“三公经费”和审计结果公开力度。制定分类推进事业单位改革实施意见，全面开展事业单位清理规范工作。加快国有企业调整重组，完成市区两级粮食企业、北控与京仪集团重组，实现骨干企业委派外部董事全覆盖，市属企业利润增长29.5%。出台促进中小企业发展的实施意见，推进中小企业公共服务平台建设，非公有制经济健康发展。加大金融创新力度，推动保障房私募债、保险资金支持土地储备等融资模式创新，促进政府融资平台规范发展，直接融资比重超过70%，上市公司数量跃居全国第二位。

　　对外开放水平不断提升。成功争取中国国际服务贸易交易会永久落户北京，制定实施建设国际商贸中心意见。地区出口增长6.5%，“双自主”企业出口比重进一步提高，服务外包增长59.3%，跨境贸易人民币结算额居全国首位。坚持市区联动、引资引智相结合，做好重大项目、总部企业投资促进工作，实际利用外资增长10.9%，新增外国企业驻京代表机构320家。建立健全境外劳务事件防范和应急处置机制，加快推动企业“走出去”，对外投资稳定增长。加强对台工作，巩固京港、京澳合作，对外经济、科技、文化交流深入推进。

　　对口支援和经济合作不断加强。投入25.2亿元，先后派出干部和专业技术人员366名，认真抓好援藏项目建设，全方位开展援疆工作，完成玉树灾后恢复重建任务的92%，对巴东、什邡的支援合作工作稳步实施，帮扶内蒙古取得新进展。首都经济圈发展研究工作全面启动，与周边省区市合作交流向纵深推进。

　　(六)加强民主法制建设，全力维护首都和谐稳定

　　认真执行市人大及其常委会的决议和决定，坚持重大事项报告制度，自觉接受人大法律监督、工作监督和人民政协民主监督，积极听取各民主党派、各人民团体和各界人士意见。办理全国和市人大代表议案、建议1442件，政协建议案、提案1385件。特别是通过办理国家文化中心建设、学前教育发展、潮白河流域综合治理、绿化隔离地区建设等4项重大议案，进一步明确了工作思路，完善了政策措施，推进了任务落实。扩大基层民主，出台全面加强城乡社区居民委员会建设意见。制定加强法治政府建设的实施意见，坚持做好政府立法工作，提请市人大常委会审议通过地方性法规6项，制定、修改、废止了11项政府规章。加强和改善行政执法，建立行政执法与刑事司法衔接工作机制，推进行政复议、应诉和调解工作，有效化解行政争议。深入开展法律服务“三进”活动，首都“六五”普法顺利启动。依法保障妇女儿童权益，出台加强孤儿保障工作意见，残疾人服务体系建设进一步加快。民族、宗教、侨务工作取得新成效。双拥共建工作深入开展，统筹城乡优抚安置保障政策，为驻京部队办好各项实事，军政军民团结进一步加强，驻京部队在首都现代化建设中发挥了重要作用。

　　确保首都安全稳定。深刻汲取安全事故教训，制定实施加强首都安全生产工作意见，全面排查治理地下空间、地下管网、地铁、特种设备安全隐患，强化重点行业安全监管，全市安全生产形势持续稳定好转，事故起数和死亡人数分别下降8%和7.4%。针对违法建设以及非法违法生产经营，深入开展专项整治行动，维护了良好的安全生产形势和社会秩序。进一步完善食品监管体系，开展打击非法添加专项行动，重点整治城乡结合部食品安全问题，加强食品生产、加工、流通、消费全过程监管，食品抽检合格率达到97.4%。坚持用群众工作统揽信访工作，大力推动领导干部接访和信访积案化解，信访总量、联名信、集体访实现三下降。加强应急协调联动，妥善处理各类突发事件。加快打造首都创新型警务，完善社会治安打防管控一体化运行机制，保持了首都安定祥和的良好局面。

　　各位代表，过去一年，我们积极应对复杂严峻的国内外经济环境，加大主动调控力度，在首钢主流程全面停产、实施房地产调控和机动车限购政策影响经济增速2个百分点的情况下，实现了平稳较快发展，取得了物价趋稳、结构优化、效益提升、民生改善的新成绩。这是党中央、国务院和中共北京市委坚强领导的结果，是全市人民齐心协力、团结奋斗的结果。在这里，我代表北京市人民政府，向全市各族人民，向人大代表、政协委员，向各民主党派、各人民团体和各界人士，向中央各部门、各兄弟省区市，向驻京解放军和武警部队官兵，向所有关心支持首都建设的港澳台同胞、海外侨胞和国际友人，表示崇高的敬意和衷心的感谢！

　　一年来，我们深入贯彻落实科学发展观，全力做好政府工作，深刻体会到，转变经济发展方式、调整经济结构，既是重大的战略任务，又是一场严峻考验，必须付出极大的努力。我们认准了方向，就要坚定信心，做好打硬仗的充分准备，坚决按照中央的部署和要求，坚持不懈地抓紧抓实抓好，努力推动首都转型发展走在全国前列。我们也清醒地认识到，首都经济社会发展仍存在不少矛盾和问题。自主创新能力还不强，培育新的经济增长点，推动产业结构优化升级任务繁重；增强发展的包容性，加强和创新社会管理，使发展成果更好地惠及广大人民群众还需加大力度；提高生态环境质量，解决好交通、人口等问题更加紧迫；生产安全、食品安全、互联网安全隐患还较多。我们要高度重视，深入分析原因，发扬敢于担当、敢于碰硬、敢于创新的精神，不断推进解决，决不辜负党和人民的期望。

　　二、2012年主要任务

　　2012年，是实施“十二五”规划承上启下的重要一年。做好2012年全市各项工作，对于巩固经济社会发展良好势头，为党的十八大召开创造良好环境，具有十分重要的意义。

　　当前，首都仍处于大有可为的重要战略机遇期。经过多年努力，转变发展方式、调整经济结构取得明显成效，服务主导、高端引领的内生动力进一步增强，科技创新、文化创新的资源优势和政策效应持续显现，各方面推动科学发展、促进社会和谐的积极性不断提高。这为做好2012年工作打下了良好基础。但是，国内外经济形势更为复杂严峻。国际金融危机的影响短期内很难消除，世界经济下行风险明显加大，复苏的长期性、艰巨性和复杂性更加凸显，外部环境对首都经济发展的不利影响仍在增加。国内经济增速放缓与物价上涨交织，一些中小企业受多重因素影响经营困难增大，经济运行的不确定性、不稳定性明显上升，保持首都经济平稳较快发展面临新的考验。我们必须全面、正确判断形势，进一步增强机遇意识、忧患意识，充分利用有利条件和积极因素，做好应对各种困难和挑战的思想和工作准备，奋力夺取首都改革开放和现代化建设的新胜利。

　　做好2012年政府工作，要全面贯彻落实党的十七大和十七届三中、四中、五中、六中全会以及中央经济工作会议精神，以邓小平理论和“三个代表”重要思想为指导，深入贯彻落实科学发展观，大力践行“北京精神”，牢牢把握“稳中求进”的总基调，加快转变经济发展方式，全力推进科技创新和文化创新“双轮驱动”，稳增长、调结构、控物价、惠民生、抓改革、促和谐，以首都科学发展、社会和谐稳定的优异成绩迎接党的十八大胜利召开。

　　2012年全市经济社会发展的主要预期目标是：地区生产总值增长8%左右；城镇登记失业率控制在2%左右；城乡居民收入实际增长7%左右；居民消费价格涨幅控制在4%左右；地方公共财政预算收入增长10%；单位地区生产总值能耗和二氧化碳排放量分别下降2.5%和3%，水耗下降3%；二氧化硫、氮氧化物、化学需氧量、氨氮排放量分别削减2%、3%、2%和2%。提出地区生产总值增长8%左右，主要是考虑了国内外复杂经济形势的影响，保持与“十二五”规划目标相衔接，引导各方面继续把工作重点放到转变发展方式、调整经济结构上来。提出城乡居民收入实际增长7%左右，主要是考虑现阶段收入增长的实际，以及物价高位运行的影响。提出居民消费价格涨幅控制在4%左右，虽然与2011年的预期目标一致，但比2011年的实际涨幅要低，有利于引导社会预期，体现稳定物价的决心。

　　2012年要重点抓好以下九个方面的工作。

　　(一)着力扩大内需特别是消费需求

　　越是在国际经济环境趋紧的时候，越是要立足于发挥首都优势，巩固扩大内需稳定增长的良好局面。

　　充分发挥消费主导作用。适应消费结构升级和变化趋势，重点围绕改善民生促消费，大力营造良好的消费环境，实现社会消费品零售额增长12%左右。一是完善便民消费体系。推动连锁企业与社区合作，提高新建社区和郊区零售网点覆盖率，丰富完善“一刻钟社区服务圈”。加快南城和新城商业设施建设，继续抓好农村消费提升改造工程。启动基本金融服务“村村通”工程，完善城乡消费金融服务，扩大消费信贷。二是加快培育新的消费热点。积极开发特色消费和品牌消费，促进“老字号”、优秀品牌振兴发展，大力发展文化、信息、健身、培训、养老、家政等服务消费，拓展网上商城、品牌直销购物中心等新兴消费业态，推动电子商务创新发展。三是充分发挥旅游对消费的带动作用。深化旅游综合改革试点，加快十大重点功能区和一批郊区旅游度假项目建设，拓展和创新旅游产品，加强旅游区域合作与国际交流。实施“五大提升工程”，大力开发休闲度假等高端旅游市场，加快推进会展业发展，深度挖掘旅游消费潜力。扩大消费的关键是增加城乡居民收入，增强居民消费能力。要落实国家收入分配改革和结构性减税政策，完善工资指导线和最低工资制度，推进工资集体协商，不断提高城乡居民特别是中低收入者的收入水平。

　　进一步优化投资结构。保持政府投资适度增长，优先安排民生保障、城市交通、资源环境、自主创新等方面投资，突出保重点、保续建、保竣工。落实鼓励引导民间投资健康发展的意见，支持社会资本进入社会事业、战略性新兴产业、市政基础设施等领域，促进民间投资稳定增长。加强投融资统筹协调，积极开展与各类金融机构对接合作，支持股权投资基金发展，做好企业上市辅导，努力提供多元化融资服务。有序安排土地开发供应，加快项目审批和开工建设。全面加强重大项目特别是政府和国有企业投资项目监管，完善工程检查、验收等机制，提高全社会投资质量和效益，实现固定资产投资增长9%左右。

　　坚定不移地抓好房地产调控。继续严格实施抑制投机投资性需求的政策措施，促进房价合理回归。完善城镇个人住房信息系统，探索促进房地产市场健康发展的长效机制。充分发挥市场力量，增加中低价位、中小套型普通商品住房供给，满足群众自住性、改善性住房需求。大力推进各类保障房建设，发挥市保障性住房建设投资中心作用，引导社会资金投入，确保新建收购16万套、竣工7万套，同步完成配套设施建设。加强公租房配租管理，做好租金测定和补贴发放工作，鼓励轮候家庭承租公租房。强化保障房后期管理服务，完善退出机制。全面完成三区三片棚户区改造任务，积极推进五片棚户区改造工作。加强房屋建设质量管理，严格责任追究，确保建成优质、放心工程。

　　继续做好稳定物价工作。以稳定食品价格、房屋租金价格为重点，深入落实物价调控各项措施，实行市与区县两级价格监控责任制，用好价格调节资金，完善市场调控预案，全面推进保供应、抓流通、重监管、稳预期等各项工作，保持物价总水平基本稳定。着力保障农副产品供应，新建3万亩基本菜田和2.5万亩设施农业，升级改造80个规模养殖场，抓好外埠生产基地建设。积极推广农副产品产销对接新模式，努力降低流通成本。完善政府储备和商业储备体系，增强市场调控能力。加强房屋租金价格指导，进一步规范房屋租赁市场。严格市场监测和价格监管，严厉查处发布虚假信息、囤积居奇、操纵价格、恶意炒作等违法行为，维护市场秩序，保护消费者利益。认真落实价格补贴政策，加强社会救助，决不能让物价上涨影响低收入群众基本生活。

　　(二)推进产业结构深度调整

　　着力培育新的经济增长点，大力发展实体经济，加快推动首都经济走上高端引领、创新驱动、绿色发展的轨道。

　　增强科技创新支撑引领作用。加大中关村示范区“1+6”政策及相关配套措施落实力度，集聚整合创新要素，着力推进科技创新和成果产业化。促进出台建设国家科技金融创新中心意见，配合建立统一监管下的全国场外交易市场。加快建设中关村科学城新兴产业技术研究院、特色产业园区，完成未来科技城主要市政基础设施和部分配套服务设施建设，推动海淀北部科技新区加快发展，促进中关村西区业态调整、环境提升。深入实施人才特区行动计划，构建机制新、活力强的人才政策体系。加强央地科研与产业资源对接，深化军民融合创新发展。完善财政资金统筹机制，重点支持对接国家科技重大专项、重大科技基础设施、重大科技成果转化和产业化项目，加快推进科技振兴产业和科技支撑民生工程。强化企业创新主体地位，建设技术创新服务平台，发挥高校院所、产业技术联盟作用，推动政产学研协同创新。深入实施知识产权和标准化战略，加强知识产权保护，认真做好专利商用化试点工作，大力发展知识产权服务业。

　　加快推动产业高端化发展。全面实施促进战略性新兴产业发展的政策措施，加大核心技术研发力度，抓好一批科技创新工程和重点项目建设，加强示范应用和市场开拓，尽快培育形成新的支柱产业。支持新一代移动通信技术研发、应用和产业化，促进物联网创新发展，加快建设中国云产业园、国际信息港、国家地理信息科技产业园、轨道交通产业园，推进三网融合试点城市建设。大力支持新能源汽车研发和产业化，加快电动汽车充换电服务网络建设，推动示范应用。深入实施生物医药产业跨越发展工程，扎实推进燕房石化新材料科技产业基地、北京通用航空产业基地、延庆新能源产业园、数字电视产业园、三一产业园等基地建设，加快培育新兴产业集群。抓好自主品牌乘用车基地、中航工业北京产业园、高端制造业房山基地等重大项目建设，促进现代制造业高端化、集约化发展。深化与中央单位战略合作，抓好已签约项目的落实，积极筛选新的后备项目，研究制定推动重大项目建设的相关政策。

　　不断提高服务业发展水平。举全市之力办好首届中国国际服务贸易交易会，着力打造“北京服务”品牌。制定实施服务业综合改革试点意见，继续抓好石景山、中关村国家服务业综合改革试点，积极准备、抓紧推进营业税改征增值税试点工作。推动新兴服务行业加快发展，做大做强信息服务业、科技服务业、商务服务业。充实完善政策，推动生活服务业转型升级。完善金融支持政策，促进要素市场规范创新发展，提高金融业发展水平。推进国际陆港等物流聚集区建设，规划建设东南农产品物流园，实施城市物流共同配送工程，促进快递、冷链物流等行业发展，增强城市物流服务保障能力。强化金融街、商务中心区、临空经济区、奥林匹克中心区的特色和功能，加快建设丽泽金融商务区、通州高端商务服务区、怀柔文化科技高端产业新区和北京科技商务区，推动形成产业发展新格局。

　　进一步推进节能减排降耗。认真落实国家下达的“十二五”时期目标任务，以内涵促降为重点，健全激励约束机制，加强考核问责，切实抓好工业、交通、建筑和居民生活等重点领域节能减排，确保完成年度任务。继续退出“三高”企业，强化对耗能耗水和污染排放重点单位的管理，全面推进清洁生产，努力实现源头和全过程控制。探索推广循环经济典型模式，抓好国家生态工业示范园区建设，大力发展新能源及节能服务业，加快资源再生利用产业化。推进住宅产业化和建筑节能改造，大力推广绿色建筑。加强对PM2.5和臭氧等重点污染物的防治，抓紧建立完善监测网络，实时发布监测信息，以更大决心、更有力措施、更高标准，实现空气质量继续改善。完成1200蒸吨燃煤锅炉清洁能源改造任务，力争淘汰15万辆老旧机动车，做好核心区非文保区1万户煤改清洁能源工作，加强工地扬尘监管，确保主要污染物浓度均下降2%。实施节能减排全民行动计划，推广应用节能节水新技术、新产品，广泛动员全社会参与节能减排。

　　(三)大力推动文化大发展大繁荣

　　充分发挥全国文化中心示范作用，以高度的文化自觉和文化自信，加快建设社会主义先进文化之都。

　　着力发展公益性文化事业。加强社会主义核心价值体系建设，大力弘扬践行“北京精神”，夯实全市人民团结奋斗的共同思想基础。扎实推进首都精神文明建设，深入开展“做文明有礼的北京人”活动，全面推进公共文明引导行动，加强改进未成年人思想道德建设和大学生思想政治教育，提高市民文明素质和城市文明程度。实施文化精品工程，设立文化艺术发展基金和首都文化贡献奖，加大文化创作支持力度。繁荣发展哲学社会科学。办好全国少数民族文艺汇演等品牌文化活动。加强文化设施规划，抓好首都核心演艺区建设，实施10项文化惠民工程，进一步完善公共文化服务体系。规范互联网信息传播秩序，发展健康向上的网络文化。

　　加快发展文化创意产业。巩固壮大传统文化产业，培育发展设计创意、动漫游戏、新媒体等新兴文化产业，协调推动文化与科技、教育、体育、旅游互动融合。加快国家广告产业园、音乐产业基地、新媒体产业园、出版创意产业园、动漫游戏城等重大项目建设，提升产业集聚发展水平。扶持优秀民营文化企业和中小文化企业加快发展，着力打造骨干文化企业。完善财税、融资等产业扶持政策，促进文化与资本深度对接。

　　加大文化体制改革力度。加快推进文化理念创新、管理创新、政策创新，促进首都文化资源整合，进一步解放和发展文化生产力。组建国有文化资产监督管理机构，设立文化创新发展专项资金，每年统筹100亿元支持文化发展。完成3家文艺院团和首批非时政类报刊出版单位转企改制。研究制定首都高端文化人才引进、培养和使用的优惠政策，完善文化人才激励机制。积极探索文化“走出去”的新途径新形式，提高首都文化的国际影响力。

　　加强历史文化名城保护。积极推动旧城整体保护，系统规划实施“一轴一线”保护整治，推进中轴线、大运河申遗工作。创新旧城街区整体保护利用机制，深入挖掘历史文化遗产的文化内涵和文化价值。完善文物保护机制，加大文物建筑腾退力度，推进文物保护由单体展示向综合保护转变。加强重大文化遗产的保护利用，推进文物保护修缮和遗址公园规划建设，抓好优秀近现代建筑和重要工业遗产的保护利用。加强对京剧、昆曲等非物质文化遗产的保护、挖掘、传承和利用。

　　(四)加大统筹城乡区域发展力度

　　坚持把做好新形势下的“三农”工作放在重中之重，进一步加大强农惠农富农政策力度，凝聚多方力量推动重点区域建设，促进城乡一体化和区域协调发展取得新突破。

　　切实提高农村经济发展水平。坚持和完善农村基本经营制度，加大耕地和基本农田保护力度，推进集体土地确权登记颁证。探索农村集体建设用地利用和管理新模式，加快区县开发区、农民就业基地建设，积极承接高端产业项目。严格落实集体土地征收制度，切实维护农民土地权益。促进新型集体经济发展，基本完成集体经济产权制度改革任务，完善集体林权制度改革配套政策，鼓励支持农民专业合作社规范发展。健全“九农”金融服务体系，提升农村金融服务水平。大力开展农田水利建设，全面完成农业基础建设及综合开发一期任务。加快国家现代农业科技城以及农业生态谷等项目建设，大幅提高农业科技投入强度，拓展都市型现代农业实现形式，加快发展高端高效农业。全面完成新农村“三起来”工程四年规划任务，做好新一轮农村电网改造和10万户农宅抗震节能改造。认真落实中央扶贫工作会议精神，对低收入农户相对集中地区给予重点扶持，加快推进农民增收致富。全力办好世界草莓大会、国际食用菌大会。

　　扎实推进重点新城和新型城镇体系建设。继续坚持聚焦通州战略，加快基础设施建设和功能区开发，推动高端商务、文化创意、医疗健康等产业发展取得新突破。积极推动顺义、亦庄—大兴、昌平和房山等其他新城建设。着力强化新城的产业功能，引导布局优质教育、卫生、文化等公共服务资源，努力提升宜居宜业水平。切实发挥小城镇发展基金引导作用，加快旅游休闲特色镇、科技和设施农业示范镇、园区经济特色镇建设，促进农村人口和产业有序聚集。完善投资机制与政策，加快郊区产业基地、园区基础设施建设。抓好新型农村社区建设试点，推动城市服务管理向农村延伸。

　　加快重点地区发展步伐。统筹做好城乡结合部重点村改制转居、就业社保等各方面工作，加快推进50个重点村建设，打造城乡一体化示范区。特别是要加强规划引导，培育发展适宜产业，抓好整体改造试点，推进工业大院“腾笼换鸟”，进一步提升产业层次。加强市级统筹协调，加快北京新机场、园博园、南中轴森林公园等重大项目建设，完成城南行动计划三年建设任务。继续推进新首钢高端产业综合服务区建设，推动永定河绿色生态发展带规划建设向南延伸，积极支持废弃矿山生态修复和替代产业发展，加快西部地区转型升级。创新沟域经济发展和经营管理模式，加强区县联合开发，促进生态型产业项目落地，打造新型山区经济带。支持密云生态商务区、门头沟旅游文化度假区建设，组织搬迁山区农民5000人，深入推进生态清洁小流域治理，提升生态涵养发展区建设水平。

　　(五)进一步加强城市服务管理

　　坚持寓管理于服务之中，加强规划引导和实施管理，促进经济社会发展与城市功能完善相协调，不断破解发展难题，提升城市承载能力。

　　加大缓解交通拥堵力度。以轨道交通建设和道路微循环改造为重点，以公交线网调整和换乘条件改善为突破口，以科技支撑和精细化管理为依托，着力提升交通服务保障能力，力争中心城区交通拥堵指数控制在6以下，公交出行比例提高到44%。加快轨道交通在建线路建设，力争分段开通4条线路。完成80项疏堵工程，打通道路微循环45公里，抓好京昆高速、京包高速、国道110二期等干道建设。推进地面公交分级建设管理，开通阜石路大容量公交，建成宋家庄、四惠等综合交通枢纽，改善公交、地铁接驳换乘条件，优化调整公交线路，提升公共交通吸引力。加强停车管理和设施建设，深入挖掘停车资源，规范整顿出租车运营秩序。建成2万辆规模的公共自行车服务系统，逐步完善步行、自行车道路系统，引导鼓励绿色出行。

　　持续改善生态环境质量。认真实施生活垃圾管理条例，加强生活垃圾全过程综合管理，加快处理设施建设，焚烧、生化处理比例分别提高到20%和30%，分类达标居住小区超过50%。提高建筑垃圾循环利用、医疗垃圾管理水平。统筹林业发展和耕地保护，通过存量建设用地腾退、废弃砂石坑绿化、二道绿隔调整种植结构、争取政策支持等措施，力争新增造林绿化面积20万亩。继续抓好城市立体绿化、宜林荒山绿化、森林健康经营、中幼林抚育等重点工作。

　　不断提高资源能源保障水平。坚持量水发展理念，实行最严格的水资源管理，建立完善用水总量、用水效率、水功能区限制纳污“三条红线”制度，开展区域、行业绩效考核。深入推进节水型社会建设，重点做好服务业和生活领域节水。加快建设南水北调市内配套工程，抓好第十水厂、郭公庄水厂等工程建设，加强水源区水资源保护，切实保障城市供水安全。推进三大水系及中小河道综合治理，加快污水处理、雨洪利用等设施建设改造，全市污水处理率达到83%，再生水利用量达到7.5亿吨。积极推进清洁能源工程，加快四大燃气热电联产项目及配套工程建设。加强能源储备和运行调控，做好高峰时段能源保障，确保供应安全可靠。

　　加强和改进城市管理。全面开展“精细管理、服务群众”活动，实施环境秩序分级分类管理，提升重点大街、重点区域、进京通道的环境景观，抓好街巷胡同和校园周边环境整治。坚持政府主导，广泛宣传发动，集中力量推进，启动全市现有882栋简易楼改造，实施1500万平方米老旧小区房屋改造工程，统筹解决好抗震节能、设施配套、停车管理等问题，力争到2015年全面完成1582个老旧小区综合改造任务。实施地下管线消隐改造，加强安全防护和动态管理。完善各类应急预案，提升监测预警指挥调度水平，提高城市抗灾应急能力。完成信息化基础设施提升计划任务，建立组织机构代码和身份证号码等为基础的实名制信息共享平台，加快建设“智慧北京”。

　　(六)着力加强以保障和改善民生为重点的社会建设

　　深入实施社会建设规划纲要，坚持积极而为，注重从各方面利益结合点上完善政策、推进改革，切实办好惠民实事，提高发展的包容性。

　　实施更加积极的就业政策。坚持就业优先战略，完善城乡统一的就业促进政策体系，加强职业培训和创业扶持，提升公共就业服务水平，实现城镇新增就业36万人，帮扶城乡8.8万名就业困难群众实现就业。进一步拓宽高校毕业生就业渠道，为就业困难群体提供精细化援助，促进农村转移劳动力稳定就业，研究制定残疾人等特殊群体就业服务政策，加大对产业转型地区稳定就业的政策支持。健全劳动关系协调合作机制，大力推行劳动合同制度，促进劳动关系和谐稳定。

　　进一步提高社会保障水平。坚持公平和适度普惠，推进社会保障由制度全覆盖迈向人群全覆盖。做好城乡结合部50个重点村农转居劳动力参保工作，统一城镇居民医疗保险报销有关政策。继续实行社会保障相关待遇标准与物价上涨挂钩联动，稳步提高社保待遇标准。加强特困人员重大疾病救助，出台慈善事业促进办法。深入落实“九养”政策，加大养老机构建设运营补贴和居家养老助残服务扶持力度，不断提升为老服务水平。

　　加快发展教育、卫生事业。优先安排教育投入，市级财政安排资金291.2亿元、增长20.7%。制定中小学建设三年行动计划，实施农村学校建设等七项工程，促进义务教育均衡发展。继续推进学前教育三年行动计划，加强幼教师资培养培训，新建改扩建50所公办幼儿园，多种形式扩大学前教育资源，新增入园名额2.4万个。深化教育教学改革，抓好名师授课数字化资源建设，加强体育、艺术、科技和实践教育，提高中小学生综合素质。严格管理、规范发展校车服务，保障学生出行安全。深入推进职业教育改革试验，启动实施高等教育质量工程和创新工程第二期项目。加强对民办教育的政策引导和规范管理。落实首都教育人才中长期发展规划纲要，加快建设高素质教育人才队伍。安排市级医疗卫生支出94.4亿元，增长25.6%。制定实施2012-2015年深化医药卫生体制改革规划，推进试点公立医院管办分开、医药分开。完善区域医疗发展规划，支持开展区域医疗合作，推动卫生信息共享，出台鼓励社会办医政策。深化家庭医生式服务，完善双向转诊机制，强化社区慢病综合管理，增强基层医疗机构服务能力。加快推进基本医疗保险付费方式改革，健全医药费用控制机制，继续减轻群众基本医疗卫生服务的经济负担。加强中医药工作，开展中医重点专科基层巡诊医疗，推进国家中医药发展综合改革试验区建设。积极开展健康促进和群众体育活动，提高市民健康水平。

　　切实加强和创新社会管理。坚持以需求为导向、服务为重点，完成500个“六型社区”示范单位和500个农村社区服务站标准化建设，在1300多个社区开展基本公共服务全覆盖试点，全面推广网格化、村庄社区化服务管理。积极推进城乡结合部、新建小区和流动人口聚居区的社区居委会组建工作。健全市、区、街三级“枢纽型”社会组织体系，创新社会组织管理方式，建立第三方评估机制，完善政府购买社会组织服务机制，引导支持社会组织开展服务民生行动。健全商务楼宇“五站合一”工作机制。加快推进社会工作者队伍专业化职业化，完善志愿服务体系。推广社区居民会议常务会、“市民劝导队”等成功经验，提高社区自治水平。加强和改进人口服务管理，深化“以业控人、以房管人、以证管人”模式，制定促进产业业态调整的专项行动计划，总结推广新居民互助服务站等经验，做好实行居住证制度的相关工作，推进实有人口服务管理全覆盖系统建设。加强人口和计划生育工作，稳定低生育水平，提高人口素质。

　　(七)坚定不移地推进改革开放

　　坚持以改革增活力、强动力，以开放促改革、促发展，不断完善有利于科学发展的体制机制。

　　积极稳妥推进重点领域改革。深化行政审批制度改革，加快市级政务服务中心建设，推动审批权限归并、下放和流程再造，扩大实行一站式审批服务。完善政府资金统筹机制，继续扩大财政预算、决算公开的范围和内容，提高预算绩效管理水平。推进绩效审计，强化审计监督。完成事业单位清理规范，推进重点领域事业单位改革先行先试。加大国有企业“调整、改革、合作、创新”力度，加快国有经济的进退调整，深化企业股份制改革，积极推进经营性国有资产证券化，加强央地、市区企业合作，增强国有企业自主创新能力，培育具有核心竞争力的大企业集团。落实促进中小企业发展的各项政策措施，构建多位一体的融资服务体系，加大创新型企业支持力度，为中小企业特别是小微企业发展营造良好环境，提升非公有制经济发展水平。加强企业信用体系建设，实现行政执法机关相关信息的统一归集、发布和运用，优化社会信用环境。认真制定方案，推进天然气价格、非居民生活垃圾收费、阶梯水价等改革。

　　加快推动外经贸转型升级。深入落实各项外贸政策，抓好口岸体系和外贸转型升级示范基地、外贸公共服务平台建设，推动天竺综合保税区、亦庄保税物流中心等外贸功能区发展，支持具有自主品牌和技术的产品出口，促进出口规模稳定增长、结构不断优化。坚持出口和进口并重，强化首都外贸枢纽功能。加强服务贸易国际国内合作，大力发展软件、生物医药等研发服务外包。健全投资促进体系，加大招商选资力度，积极吸引跨国公司地区总部，推动研发中心、采购中心等功能性机构落地。支持优势企业开展技术合作、商业服务等境外投资。积极吸引国际组织落户，争取更多有影响力的国际活动在京举办，深化对外务实交流合作。进一步拓展京台、京港、京澳合作领域，做好侨务工作。

　　进一步做好对口支援和经济合作。认真落实对口支援和对口帮扶各项规划，坚持首善标准，全面完成年度各项援建任务。深入推进援藏、援疆工作，扎实做好内蒙古对口帮扶，完成玉树灾后恢复重建任务。建立首都经济圈协调发展机制，落实与兄弟省区市合作协议，加强生态建设、污染治理、交通规划、城镇化发展、产业布局等方面的统筹协调，推动区域合作取得新成效。

　　(八)巩固发展首都和谐稳定的良好局面

　　不断加强民主法制建设。继续推进村务公开、厂务公开，推动村务监督委员会规范化建设，保障群众民主管理、民主监督的权利。研究制订村民委员会组织法实施办法和村民委员会选举办法，依法组织第八届社区居委会选举。加快法治政府建设，完善重大行政决策程序，重点做好经济发展方式转变、国家文化中心建设、社会管理创新、保障改善民生、资源和生态保护等方面的立法工作，完善行政执法与刑事司法衔接工作机制。做好“六五”普法工作，加强公益法律服务。实施妇女儿童发展规划。加强民族、宗教政策宣传，健全民族特色服务体系，依法管理宗教事务。认真落实保障残疾人合法权益的政策法规，继续推进残疾人服务体系建设，完成100个住宅小区无障碍改造任务，丰富残疾人精神文化生活。积极拓展双拥共建工作内涵，加强全民国防教育和国防后备力量建设，提升优抚安置保障水平，巩固和发展军政军民团结。

　　深入推进“平安北京”建设。健全重大事项社会稳定风险评估机制，推广用群众工作统揽信访工作试点经验，深入开展矛盾纠纷排查调处活动，完善大调解工作体系，千方百计化解信访积案，千方百计减少新的矛盾，努力把矛盾纠纷解决在基层和萌芽状态，坚决防止发生影响社会稳定的重大信访问题和群体性事件。完善安全监管长效机制，建立安全生产责任可追溯制度和责任保险制度，全面推行安全生产标准化，大力开展企业职工安全培训，加强特种设备安全监察管理，严厉打击非法违法生产经营建设行为，切实维护人民群众生命财产安全。建立健全食品、药品市场准入和退出的强制性标准体系，持续开展专项整治行动，加强监管能力建设，落实监管责任，促进行业自律，确保群众吃得放心、用药安全。深化社会治安防控体系建设，严厉打击各类违法犯罪活动，不断完善区域警务合作和群防群治机制，全面强化专项安保工作，切实营造和谐稳定的社会环境。

　　(九)全面加强政府自身建设

　　过去一年，我们深入开展创先争优活动，深化行政管理体制改革，精简行政审批事项，强化政府绩效管理和行政监察，加大源头反腐工作力度，政府自身建设进一步加强。但是，我们也清醒地看到，政府工作与广大人民群众的期望还存在差距，必须进一步转变政府职能，加快服务管理创新，切实提高行政效能，不断增强公务员的责任意识、服务意识，更好地适应新阶段、新形势、新任务的要求，全面、正确、高效履行政府职能。

　　在新的一年里，我们将坚决执行市人大及其常委会的决议和决定，自觉接受人大法律监督和工作监督，自觉接受人民政协的民主监督，认真听取各民主党派、各人民团体和各界人士的意见建议，带头践行“北京精神”，深入推进科学民主决策和依法行政，把“十二五”规划的主题主线、目标任务、战略举措充分体现到工作中去。

　　我们将着力转变政府职能，更加重视社会管理和公共服务，加快健全覆盖城乡的公共服务体系，全面增强基本公共服务能力。继续深化行政管理体制改革，大力推进服务型政府建设，努力为各类市场主体创造公平的发展环境，为人民群众提供良好的公共服务，维护社会公平正义。

　　我们将切实加强政府作风建设，深入推进政务公开，加强绩效管理、效能监察和行政问责。大兴求真务实之风、艰苦奋斗之风，继续精简会议和文件，集中精力把定下来的事情一抓到底，坚持勤俭节约办一切事情。加强公务员教育和管理，规范行政执法，努力提供更加规范有序、高效便民、公开透明的政府服务。

　　我们将深入开展廉政建设和反腐败斗争，加强廉政文化建设，推进廉政风险防控管理，健全行政权力运行制约和监督机制，严格执行廉政建设责任制，坚决纠正损害群众利益的不正之风，严肃查处各类违纪违法案件，加快建设廉洁政府。

　　各位代表，首都北京正迈步在科学发展的康庄大道上，任何艰难险阻都挡不住我们前进的步伐。让我们更加紧密地团结在以胡锦涛同志为总书记的党中央周围，高举中国特色社会主义伟大旗帜，在中共北京市委坚强领导下，紧紧依靠全市各族人民，坚定信心，砥砺奋进，全面实施“人文北京、科技北京、绿色北京”战略，把中国特色世界城市建设奋力推向前进！
